# Walk-ins seeking treatment at an emergency department or general practitioner out-of-hours service: a cross-sectional comparison

**DOI:** 10.1186/1472-6963-11-94

**Published:** 2011-05-09

**Authors:** Corinne Chmiel, Carola A Huber, Thomas Rosemann, Marco Zoller, Klaus Eichler, Patrick Sidler, Oliver Senn

**Affiliations:** 1 Institute of General Practice and Health Services Research, University of Zurich, Zurich, Switzerland; 2 Institute of Health Economics, Zurich University of Applied Sciences, Winterthur, Switzerland; 3 Emergency Department, Waid City Hospital, Zurich, Switzerland

## Abstract

**Background:**

Emergency Departments (ED) in Switzerland are faced with increasing numbers of patients seeking non-urgent treatment. The high rate of walks-ins with conditions that may be treated in primary care has led to suggestions that those patients would best cared for in a community setting rather than in a hospital. Efficient reorganisation of emergency care tailored to patients needs requires information on the patient populations using the various emergency services currently available. The aim of this study is to evaluate the differences between the characteristics of walk-in patients seeking treatment at an ED and those of patients who use traditional out-of-hours GP (General Practitioner) services provided by a GP-Cooperative (GP-C).

**Methods:**

In 2007 and 2009 data was collected covering all consecutive patient-doctor encounters at the ED of a hospital and all those occurring as a result of contacting a GP-C over two evaluation periods of one month each. Comparison was made between a GP-C and the ED of the Waid City Hospital in Zurich. Patient characteristics, time and source of referral, diagnostic interventions and mode of discharge were evaluated. Medical problems were classified according to the International Classification of Primary Care (ICPC-2). Patient characteristics were compared using non-parametric tests and multiple logistic regression analysis was applied to investigate independent determinants for contacting a GP-C or an ED.

**Results:**

Overall a total of 2974 patient encounters were recorded. 1901 encounters were walk-ins and underwent further analysis (ED 1133, GP-C 768). Patients consulting the GP-C were significantly older (58.9 vs. 43.8 years), more often female (63.5 vs. 46.9%) and presented with non-injury related medical problems (93 vs. 55.6%) in comparison with patients at the ED. Independent determining factors for ED consultation were injury, male gender and younger age. Walk-in distribution in both settings was equal over a period of 24 hours and most common during daytime hours (65%).

Outpatient care was predominant in both settings but significantly more so at the GP-C (79.9 vs. 85.7%).

**Conclusions:**

We observed substantial differences between the two emergency settings in a non gate-keeping health care system. Knowledge of the distribution of diagnoses, their therapy, of diagnostic measures and of the factors which determine the patients' choice of the ED or the GP-C is essential for the efficient allocation of resources and the reduction of costs.

## Background

In most industrialised countries the number of patients seeking non-urgent care at EDs (emergency departments) is increasing immensely [1-6]. In hospitals in Zurich, Switzerland, the number of emergency medical encounters has doubled in the last 10 years with an annual growth rate of 2-8% [7,8]. Literature reports an enormous variability (6-80%) on the percentage of walk-ins to EDs who could have been treated by a GP (general practitioners) [9-16]. According to the data available, the vast majority of these walk-in patients were able to be treated as outpatients [2,6-8,16-23] and hospitalisation was rarely necessary.

In Switzerland, patients generally have unlimited access to the health care system. Patients seeking emergency care can contact either their own GP, a GP-C (General Practitioner Cooperative) providing out-of-hours emergency services, sporadic urban walk-in emergency centres, or a hospital ED. Access to these treatment options is unrestricted and mandatory health insurance covers all costs (except for basic annual deductibles of between 300 and 2500 Francs and patient payment of 10% of all costs), regardless of which service is used. Unlike other European countries, there are no barriers (gate-keeping systems). In Switzerland, particularly in urban areas and during nights or weekends, EDs are often used as substitutes for primary care physicians and this has resulted in an increase in the walk-in burden placed on hospital EDs. Different reasons for the growing demand for emergency consultations can be found. On the one hand, the aging of the population, which is associated with an increase in chronic diseases and multimorbidity leads to a general increase in consultation numbers [24,25]. On the other hand non-health related factors often affect decisions to seek treatment in an ED rather than in a primary care service [11,26-28]. Among younger patients it has become increasingly common not to have a personal GP and to show consumer behaviour towards health care services. Furthermore, patients tend to perceive hospitals as centres of competence with a broader spectrum of expertise and more technical resources. The reason most frequently cited by patients for by-passing GP care providers is the belief that radiography is necessary [12,14,15,29].

The congestion of EDs by patients seeking non-urgent medical treatment is disadvantageous to both patients and staff and increases health care costs. In an attempt to redress this situation many hospitals have undergone restructuring and a variety of organisational models designed to reduce inappropriate use of EDs [18-20,30-35] have evolved. In the greater Zurich area, for example, several hospitals have established primary care centres as an integral part of their EDs.

If emergency care is to be effectively reorganised into a system tailored to patient needs it is essential to know whether patients consulting the EDs are different from those using the traditional out-of-hours services provided by a GP-C as far as diagnosis, diagnostic procedures and therapy are concerned [3,19,23,26-28,36-39]. International data indicates that there are differences between the diagnoses of EDs and those of GP out-of-hours services. However, there is only scant data on health care systems without gate-keeping functions and very little specifically Swiss data [40]. The aim of the study is to compare the characteristics of walk-in patients at an ED with those of patients who use the traditional out-of-hours service provided by a GP-C.

This study demonstrates the initial results of an ongoing evaluation of the effects of integrating a primary care service run by GPs into the ED at the Waid City Hospital in Zurich, with one access point to medical care. This project will show whether a change in the system can reduce the burden of walk-in patients with its negative consequences for an ED.

## Methods

### Setting

In the city of Zurich (population 400,000) the out-of-hours-service of the GP-C is currently organised by an Emergency Medical Service Telephone (EMST) Switchboard, which is a unit of the general emergency medical service [39]. The features of the GP-C can be seen in Appendix 1. The reasons for choosing this service in preference to the patient's own GP vary. Patients either do not have a GP or deliberately do not want to consult their GP, the GP might be absent or occupied or the emergency occurs outside of practice opening hours. After contacting the EMST patients are guided to the GP on duty. GPs have a mandatory rota system providing a 24/7 out-of-hours service with shifts lasting from 7 a.m. to 7.00 a.m. the following day. For each of the five Zurich emergency service areas there is one GP on duty. Between 10.00 p.m. and 7.00 a.m. a so-called night doctor primarily provides out-of-hours care and the GP is on back-up service. The night doctor provides only telephone consultation and home visits, whereas GPs also provide practice consultations. Our study covered all GP and night doctor patient encounters connected via the EMST during a 24-hour service period. Concomitantly the same evaluations were performed in the ED of the Waid City Hospital in Zurich.

### Subjects, data collection and measurements

**Figure 1:**
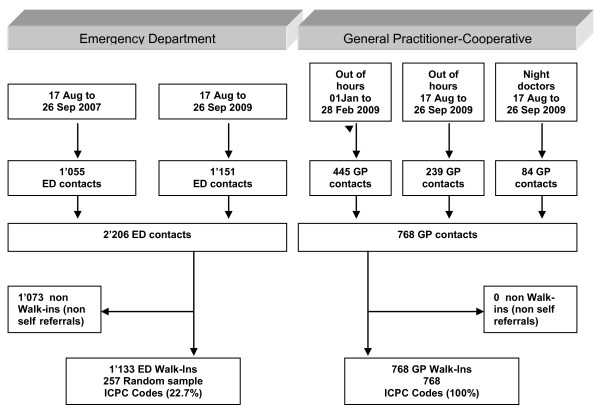
**Study flow**. Study flow of different phases of data collection at the ED as well as GP-C from August 17 2007 until September 26 2009. In total 2206 ED and 768 GP-C encounters were registered. 1073 of the ED encounters were non walk-ins. All the patients consulting the GP-C were walk-ins. Out of the 1133 walk-ins at the ED a random sample of 257 (22.7%) encounters were coded according to ICPC. All of the 768 (100%) encounters in the GP-C were coded.

Our study covered two time periods (summer and winter) to take into account seasonal variability of diseases. Outcomes were compared between the out-of-hours GP-C service and the ED at the Waid City Hospital, with special emphasis on walk-in patients. The detailed study flow can be seen in Figure 1.

The data on the out-of-hours service was collected between 1^st ^January and 28^th ^February, 2009 and 17^th ^August and 26^th ^September, 2009. During the period from 17^th ^August to 26^th ^September, 2009 additional data on the night doctor was collected. Questionnaires were sent by the EMST switchboard via email to all on-duty GPs. Before the beginning of a duty period, the EMST called the GP on duty in order to ensure that he had received the questionnaire and was aware of the study. If data was not provided by two days after the duty period a further telephone reminder was conducted by the EMST. The GPs documented time, number and mode of encounter (home, practice or telephone contact), as well as patient variables (age, gender) and medical problems. Diagnostic procedures and the mode of care subsequent to the emergency encounter (e.g. final treatment by GP defined as outpatient care or transferral to hospital) were recorded.

Data on the ED at the Waid City Hospital was collected on all patients admitted consecutively between 17^th ^August and 26^th ^September, 2007 and 17^th ^August and 26^th ^September, 2009. Time intervals of emergency care, source of referral, medical problems, diagnostic procedures, and mode of discharge after ED treatment were assessed by a validated outcome tool ("emerge") [8].

### Processing and analysing data

During the duty period of the GPs, patient characteristics were documented for the first, second and the last patient encounter. During the out-of-hours service of the night doctors they were documented for the first, second, third and the last patient encounter. Medical problems for all documented patient encounters were coded by one research assistant according to International Classification of Primary Care, ICPC-2 [41] and data was entered by an independent assistant at the Institute of General Practice and Health Services Research. Data sheets at the Waid City Hospital were completed by the various staff members directly involved in patient care. Processing of the raw data was performed by the "Verein Outcome", a non-profit public data processing organisation responsible for quality control measurements in health care [8], and returned on file. A random sample of 22.7% of the walk-ins (n = 257) was given at least one ICPC-2 code. To ensure data validity the intra-rater reliability for the repeated coding of a random sample of 130 diagnoses according to ICPC-2 was computed. It was high with a Cohen's Kappa ranging from 0.88 to 0.96 on chapter, component und diagnosis level.

Data was checked for eligibility and completeness and subjected to a set of predefined plausibility tests. These included checks for contradictory data, duplication and plausibility of time measurements.

### Statistical analysis

Continuous variables were summarised as medians/interquartile ranges and categorical data as frequencies. We compared patient characteristics between the two settings using non parametric tests. The level of significance was set at 0.05. To investigate independent determinants for contacting GP-C or ED we applied multiple logistic regression analysis. The independent variables were gender, age, walk-in time and diagnosis. All analyses were calculated using the STATA statistical package, version 10.1 (Stata Incorporation, College Station, TX, USA).

### Ethics approval

Approval of the study was given by the Ethics Committee of the Canton of Zurich (reference Nr. 26/09).

## Results

100% of the consecutive consultations at the ED were documented. 52% of the questionnaires from the GP-C consultations were returned. During the various phases of data collection a total of 2974 encounters were registered (ED summer 2007: 1055 encounters; ED summer 2009: 1151 encounters; GP-C winter 2009: 445 encounters; GP-C summer 2009: 239 encounters; night doctors summer 2009: 84 encounters) (Figure 1). At the GP-C 100% of the encounters were walk-ins. At the ED 54.0% in 2007 and 48.9% in 2009 were walk-ins (p = 0.016 for the difference in the number of walk-ins) and underwent further analysis, resulting in a total of 1901 encounters (Figure 1 and Table 1).

**Table 1:** Patient characteristics by evaluation period

	Emergency Department		General Practitioner-Cooperative		
	**Summer 2007 ** **(n = 570)**	**Summer 2009 ** **(n = 563)**	**GPs** **Winter 2009** **(N = 445)**	**GPs** **Summer 2009** **(N = 239)**	**Night doctors** **Summer 2009** **(n = 84)**

Age (years)	44.4 (42.6-46.1)	43.2 (41.4-44.9)	58.8 (56.3-61.2)	59.6 (55.8-63.4)	58.1 (52.4-63.8)

Male (%)	52.5	53.8	35.0	- °	43.9

Walk-in time (%)					
7-19	65.6	61.6	69.5	74.2	NA ^#^
19-22	17.7	13.9	20.2	19.1	NA ^#^
22-7	16.7	24.5 *	27.0	13.0	100.0

Mode of contact (%)					
Practice cons.	NA	NA	26.8	22.7	NA ^#^
Home visit	NA	NA	59.5	63.1	95.9
Telephone	NA	NA	13.7	14.1	4.1

* p < 0.01 Versus ED summer 2007 (22-7)

° In the GP-C in summer 2009 no data were collected on gender

^# ^Not Applicable: night doctors only consult from 22.00 p.m. -7 a.m. and do not offer practice consultations

Table 1 shows that the distribution of patient age and gender did not differ between the ED evaluations in 2007 and 2009. Similarly no difference was noted in the age and gender distribution of GP-C or night doctor patients in the winter and summer of 2009. Distribution of walk-in times to the GP-C did not differ in the different evaluation periods. Walk-in patients showed up in both settings mainly during the daytime (61.6-74.2%).

There was a significant increase in walk-ins at night in 2009 compared to 2007 (p < 0.01).

At the GP-C the demand for home visits was significantly higher than that for practice and telephone consultations (in total 63% vs. 23.5 and 13.1%). No seasonal variation between the observed modes of contact at the GP-C could be found.

Considering all of these findings, the data was pooled for further analysis as presented in Table 2.

**Table 2:** Patient characteristics and treatment of pooled evaluation periods

	Pooled (n = 1133) ED summer 2007 ED summer 2009	Pooled (n = 768) GP-C winter 2009 GP-C summer 2009 Night doctors summer 2009
Age (years) *	43.8 (42.5-45.0)	58.9 (57.0-60.8)

Male (%)*	53.1	36.5

Walk-in time (%)		
7-19	63.6	60.4
19-22	15.8	16.7
22-7	20.6	23.0

Diagnostics (%) *		
No	22.3	80.7
Laboratory analysis	54.8	15.3
Radiography	45.3	1.2
EKG	23.2	1.7
Sonography	5.9	0.5
Other °	12.3	2.8

Outpatient care (%)*	79.9	85.7

Injury (%)* ^#^	44.4	7.0

* p < 0.05 between the ED and GP-C

° ED: CT, Specialist Consultation, Duplex-Sonography, Echocardiography, Interventional Radiology, MRI, Endoscopy (detailed frequencies see text)

^# ^According to the ICPC components patients were classified into injury versus non-injury related medical problems

### Comparisons between ED walk-ins and GP-C out-of hours services (Pooled Data)

Walk-in patients from the GP-C were significantly older (58.9 years versus 43.8 years) and significantly more often female (63.5 versus 46.9%), compared to patients in the ED (p < 0.01 for both). Patients of the GP-C underwent significantly fewer diagnostic tests (p < 0.01) than the walk-ins at the ED. The most commonly performed tests in both settings were laboratory analysis (54.8% and 15.3%). Walk-ins at the ED were significantly more likely to receive conventional radiography, electrocardiography, sonography and other tests. Other tests at the ED were related to CT scan (6.2%), specialist consultation (3.2%), duplex-sonography (0.6%), echocardiography (0.4%), interventional radiology (0.3%), MRI (0.1%) and Endoscopy (0.1%). The remaining 2.5% "other tests" at the GP-C were not further specified. In both settings the majority of consultations could be resolved by outpatient care and hospitalisation was not necessary (79.9% at the ED and 85.7% at the GP-C (p < 0.01)).

To exclude potential confounding due to seasonal variation we performed additional analysis restricted to summer evaluation periods (i.e. ED vs. GP-C summer periods). The predominance of male patients at the ED persisted but did not reach statistical significance (53.1 vs. 43.9%). Significantly more walk-ins occurred during night time at the GP-C in the summer periods as compared to the ED (34.8 vs. 20.6%). However, daytime consultations were persistently predominant. The distribution of diagnostics, outpatient care and injury did not differ between the stratified summer and the overall analysis.

Overall a wide range of problems classified according to the ICPC-2 (131 at the ED and 163 at the GP-C) could be observed. Out of the 163 different diagnoses at the GP-C only 4 diagnoses showed a frequency of more than 5%. Out of the 131 different diagnoses at the ED only two showed a frequency of more than 5%. At the GP-C 40 different diagnoses surpassed the threshold of a frequency of 1% related to all diagnoses, at the ED 26 diagnoses surpassed the prevalence threshold. These 40 and 26 diagnoses represented only 24.5% and 19.8% of all encounter reasons. 75.5% to 80% of the diagnoses represented relatively rare conditions (<1 per 100 patient encounters).

**Figure 2:**
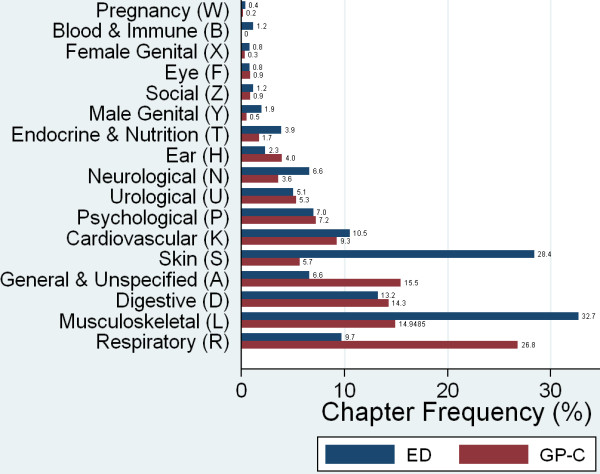
**Distribution of ICPC chapters presenting at the ED or GP-C**. The GP-C dealt mainly with problems related to respiratory (Chapter R) and general complaints (Chapter A) (26.8% and 15.5%), as well as with musculoskeletal problems and gastrointestinal infections (Chapter L and D) (15.0 and 14.3%). Musculoskeletal- (Chapter L) and skin related problems (chapter S) were most common in walk-ins at the ED with a prevalence of 32.7%, and 28.4%, respectively.

**Figure 3:**
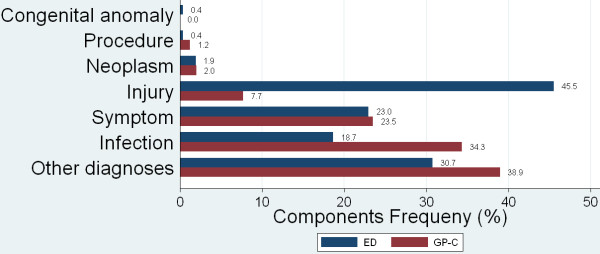
**Distribution of ICPC components presenting at the ED or GP-C**. Injuries related to the musculoskeletal system and the skin (Chapter L and S) were the most common diagnoses in ED walk-in patients (45.5%). At the GP-C Infections (34.3%) and other diagnoses (38.9%) predominated.

Figure 2 and 3 show the distributions of chapters and components compared between the ED and GP-C. Injuries related to the musculoskeletal system and the skin (Chapter L and S) were the most common diagnoses in ED walk-in patients (32.7% and 28.4%). The GP-C dealt mainly with respiratory problems (Chapter R) and general complaints (Chapter A) (26.8% and 15.5%), as well as with musculoskeletal problems and gastrointestinal infections (Chapter L and D) (15.0% and 14.3%).

The top five diagnoses showed clear differences between the ED and GP-C (Table 3). The GP-C was most commonly confronted with influenza (7.3%), followed by back syndrome (6.7%), acute upper respiratory infection (5.4%), gastroenteritis (5.4%) and cystitis (3.3%). Walk-ins at the ED mainly presented with the diagnoses laceration (13.2%), contusion (7.8%), back syndrome (5.8%), sprain of ankle (4.7%) and fracture of hand or foot (4.7%).

**Table 3:** The most frequently presented problems at the ED and GP-C pooled overall

ED Pooled (n = 1133)			GP-C Pooled (n = 768)		
Diagnosis	ICPC	Frequency (%)	Diagnosis	ICPC	Frequency (%)
Laceration/cut	S18	13.2	Influenza	R80	7.3

Bruise/contusion	S16	7.8	Back syndrome w/o radiating pain	L84	6.7

Back syndrome w/o radiating pain	L84	5.8	Upper respiratory infection acute	R74	5.4

Sprain/strain of ankle	L77	4.7	Gastroenteritis pre- sumed infection	D73	5.4

Fracture hand/foot bone	L74	4.7	Cystitis/urinary infection other	U71	3.3

**Figure 4:**
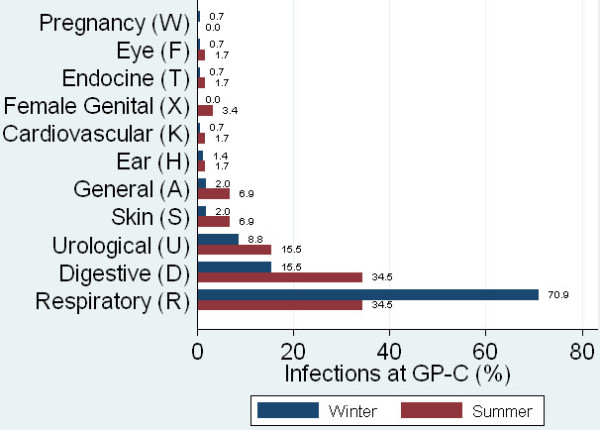
**Seasonal distribution of Infections at the GP-C**. Infection related problems presented in the GP-C showed seasonal variation with regard to the affected organ system. In winter, infections of the respiratory system were more common (70.9%), in summer gastrointestinal infections (34.5%) predominated

The medical problems presented to the GP-C showed seasonal differences especially within the chapter infections. In winter, infections of the respiratory system were more common (70.9%), in summer gastrointestinal infections (34.5%) predominated (Figure 4). In the stratified analyses for summer evaluation periods the diagnoses associated with respiratory infections were replaced by gastroenteritis (9.6%), hypertension (4.9%) and vertigo (3.0%). The prevalence of back syndrome (6.4%) and cystitis (3.8%) did not differ before and after stratification for season (Table 4).

**Table 4:** The most frequently presented problems at the ED and GP-C pooled for summer evaluation periods

ED Pooled (n = 1133)			GP-C-Summer Pooled (n = 323)		
Diagnosis	ICPC (%)	Frequency	Diagnosis	ICPC (%)	Frequency
Laceration/cut	S18	13.2	Gastroenteritis pre- sumed infection	D73	9.6

Bruise/contusion	S16	7.8	Back syndrome w/o radiating pain	L84	6.4

Back syndrome w/o radiating pain	L84	5.8	Hypertension uncomplicated	K86	4.9

Sprain/strain of ankle	L77	4.7	Cystitis/urinary infection other	U71	3.8

Fracture hand/foot bone	L74	4.7	Vertiginous syndrome	H82	3.0

**Figure 5:**
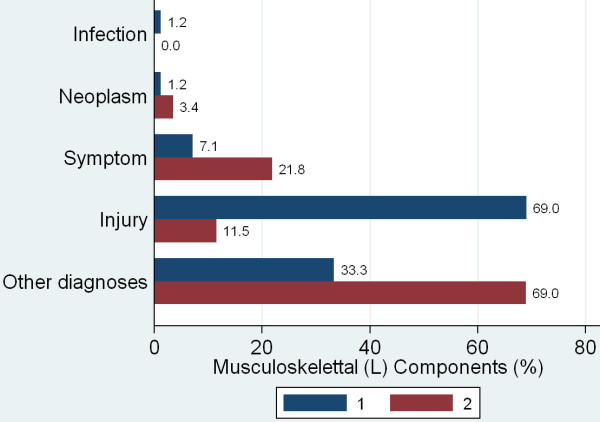
**Musculoskeletal problems, distribution at the ED or GP-C**. Musculoskeletal problems (chapter L) were frequent at the ED as well as the GP-C. At the ED the diagnose was mainly due to the component injury (69.0%), at the GP-C mainly due to component other diagnoses (69.0%), mostly comprised of lower back pain.

The chapter L (musculoskeletal) was frequent in the ED (31.5%) as well as the GP-C (15%). At the ED, the musculoskeletal problems were mainly injury related (69.0%), whereas at the GP-C non-injury related low back pain (ICPC component "other diagnoses" 69.0%) was dominant (Figure 5).

We further analysed determinants which were associated with patients' decisions to choose either the ED or GP-C for consultation. Younger age, male gender and injury-related medical problems remained independently associated with walk-ins to the ED when additionally controlled for walk-in. Corresponding odds ratios were 0.99 for age (years) (95 CI 0.98-0.99), 1.7 for male gender (95 CI 1.1-2.6) and 14.2 for injury (95 CI 7.4-27.1).The total explained variance for healthcare utilisation was 33% (Nagelkerke test R^2 ^= 0.33).

## Discussion

Knowledge of the distribution of diagnoses, the related therapy, of diagnostic measures and of the factors which determine the patients' choice of emergency care services is essential for the efficient allocation of scarce health care resources. Our study provided detailed information on walk-in patients who consulted either an ED or a GP-C and their medical problems. We observed substantial differences between the two primary care emergency settings.

### Patient demographics (age and gender)

Patients consulting the GP-C were significantly older, consistent with data found in literature [3,23,37]. They were also significantly more often female in comparison to patients at the ED. In literature it has been observed that, in general, women show a higher utilisation of the health care system, usually explained by differences in health seeking behaviour, itself explained by differences in social role, health knowledge, health status, sensitivity to symptoms, willingness to report health problems, acceptance of help seeking, compliance with treatment [3,23,37,42-48]. The reasons for the preference of consulting the GP-C over the ED are not known and further analysis is necessary.

### Outpatient care versus hospitalisation

Outpatient care was predominant in both settings but significantly more common in the GP-C.

About 50% of the patients consulting the ED were walk-ins and the vast majority of these patients could be treated as outpatients. This high proportion of walk-in patients seeking non-urgent care demonstrates the burden hospitals are confronted with [1-6].

The rate of home visits at the GP-C was high (63%), suggesting that the ED may not cover all the patient demands [31,34] as already described by Huber et al [39].

### Walk-in times

No differences could be found in the distribution of walk-ins over the daytime. Patients mainly presented from 7.00 a.m. to 7.00 p.m. (about 65%), which is in accordance with data found in literature [49].

### Medical problems and diagnostics

The distributions of diagnoses differed between the settings, which is in line with findings reported in different health care systems [16,17]. Injuries were the most common diagnoses in ED walk-in patients (45.5%), an observation which is in accordance with literature [3,23,37]. The GP-C dealt mainly with respiratory problems and general complaints (26.8% and 15.5%), as well as with musculoskeletal problems and gastrointestinal infections (15.0% and 14.3%).

Both settings showed a broad spectrum of medical problems, which is a typical observation for the primary care setting [3,24,50]. At the GP-C there was a broader distribution of medical problems [39]. The most common problems were infections (in winter respiratory, in summer gastrointestinal), musculoskeletal problems (especially low back pain) and other problems (mainly limited function/disability, fainting, unspecified viral disease and fever).

In our study significantly more radiological diagnostics were applied at the hospital. The reason for this difference is multifaceted. The differences in diagnoses with a peak in injury-related medical problems suggest a correlation with the rate of (radiological) diagnostic measures. In order to evaluate appropriateness of the diagnostic measures correctly, further aspects such as doctor and patient behaviour and differences in health care settings have to be taken into account. Earlier studies have demonstrated a decrease in the use of additional diagnostics when GPs were on duty at the ED [12,22,30,51]. Kulu et al. [29] and Sempere-Selva [52] had observed, that patients bypass the GP due to the belief that GPs lack technical resources. Finally, the lower number of diagnostic tests at the GP-C can also be explained by the high rate of home visits, where diagnostic testing is limited. The fact that patient age and gender differ between the two settings further complicates a direct comparison.

### Determinants of choosing a specific emergency care setting

Both younger age and male gender are independent predictors for choosing the ED, when controlled for injury-related medical problems, showing that injury alone does not explain the difference in health care utilisation. Other studies have shown that often non-health related, mainly socioeconomic, factors affect decisions to seek treatment in an ED rather than in primary care [11,19,20,26-28,53].

### Strengths and limitations

A limitation is that data collection in a winter period could only be undertaken at the GP-C with no parallel period at the ED. This was due to the fact that the evaluation periods at the ED had to be coordinated with the "emerge" measurements [8]. The effect on results is probably small because the known seasonal variation in diagnoses, particularly where infections are concerned, is likely to affect the ED and the GP-C similarly [49]. This assumption is reinforced by our stratified analysis for the summer evaluation periods. Diagnoses associated with respiratory infections were replaced by gastroenteritis. The prevalence of back syndrome and cystitis did not differ before and after stratification for season.

Medical problems were assessed according to ICPC, a system especially designed and validated for the primary care setting [41] and a high intra-rater reliability of the ICPC codings could be found. Diagnoses from the ED were coded for a random sample of 22.7% due to feasibility reasons, and showed morbidity rates comparable with previous studies in similar settings [3,10-12,37], suggesting that the randomisation was representative for the whole collective. Our data collection was based on a validated benchmarking tool "emerge" [8], which was developed for quality control purposes for Swiss hospitals. The Waid City Hospital also participated in the evaluation study of the "emerge" tool in which it showed no significant differences compared to the other hospitals included in the study. It can thus be stated that the data from the Waid City Hospital is representative for other Swiss hospitals, despite the study being limited to an urban setting. The mandatory GP rota system for out-of-hours services is common in both rural and urban areas. We are also of the opinion that the GP out-of-hours service mix for our urban sample (i.e. little diagnostic testing and basic care) applies to rural areas at least as well.

Our study was undertaken prospectively in two different real-world emergency care settings providing detailed patient characteristics with an emphasis on walk-in patients. In both settings the participation rate was very high with 100% at the ED and 52% at the GP-C, which is higher than expected when dealing with GP surveys [54].

### Implications for health service research and policy decision makers

Similar studies have been performed in other (European) countries [23,33,37,55]. The main difference when comparing these with our study is related to the health care setting. In Switzerland no gate-keeping framework exists and access to any kind of emergency medical facility is covered by mandatory health insurance (except for basic annual deductibles of between 300 and 2500 Francs and patient payment of 10% of all costs). For this reason comparison between countries is limited and optimisation of the allocation of resources in emergency care would depend on health-care system specific data [56]. In our non gate-keeping setting, walk-ins at the ED showed a broad and low prevalent distribution of diagnoses, comparable to other primary care settings. This gives rise to the suggestion that GPs be brought to where patients go. This approach seems, at least in the short term, to be a more practical way of dealing with the walk-in burden at the EDs than the reorganisation of the entire health care system. This study is part of an ongoing evaluation of the implementation of a general practice integrated into a hospital ED in Zurich [39,56]. Its aim is to investigate the effects on quality of care and the economic consequences of a hospital-based general practice with one access point for patients. The results of this project contribute valuable information for service planning [57,58] especially for countries without gate-keeping systems such as Germany, Belgium [31] or the US [59].

## Conclusions

Our study showed that walk-ins seeking emergency care at a GP-C or ED presented with differing problems, which were nevertheless typical for primary care. Countries with no gate-keeping system have difficulties redirecting patients streaming to EDs. A possible solution to this problem might be the integration of a primary care centre into a hospital ED. Policy makers should be interested in the potential to increase the quality of care and to optimize the allocation of limited resources, which could be achieved by close collaboration between different providers of emergency care.

## List of Abbreviations

(GP-C): General Practitioner-Cooperative; (GP): General Practitioners; (ED): Emergency Department; (ICPC): International Classification of Primary Care.

## Competing interests

The authors declare that they have no competing interests.

The study was supported by a project fund of the Health Department of the City of Zurich, Switzerland. The funding source had no influence on study design on the collection, analysis, and interpretation of the data on the writing of the manuscript and the decision to submit the manuscript for publication.

## Authors' contributions

CC was a study investigator and wrote the drafts of the manuscript. CC and OS performed statistical analysis and interpreted data. OS was a study investigator and substantially contributed to and reviewed the drafts of the manuscript. CAH, TR, MZ and KE developed the study protocol and were study investigators. PS was responsible for data collection and organisation of evaluation periods at the ED. All authors reviewed drafts of the manuscript, read and approved the final manuscript.

## Appendix 1

Features of general practice cooperatives (GP-C) in Zurich

○ Access via single regional telephone number: Emergency Medical Service Telephone Switchboard (EMTS)

○ EMTS guides patient to GP or night doctor on duty

○ Access 24/7

○ Between 10 p.m. and 7 a.m. the night doctor primarily provides the out-of-hours-care and the GP is on back-up service

○ From 7 a.m. to 10 p.m. telephone consultations, home visits and practice consultations provided by GP

○ From 10 p.m. to 7 a.m. telephone consultations and home visits provided by night doctor

○ Doctors on duty situated throughout the city, with one GP on duty for each of five Zurich emergency service areas

○ Handling of about 80'000 patients within a diameter of 7-12 km

○ Home visits until 10 p.m. using a fully equipped private GP car (with for example oxygen, intravenous drip, automatic defibrillation equipment)

○ Home visits from 10 p.m. to 7 a.m. using a fully equipped recognisable night-doctor's car

## Pre-publication history

The pre-publication history for this paper can be accessed here:

http://www.biomedcentral.com/1472-6963/11/94/prepub
